# Influence of the Climate of Peru on Pulmonary Consumption

**Published:** 1856-12

**Authors:** Archibald Smith


From the Medico-Chirurgical Review for October.

*Influence of the Climate of Peru on Pulmonary Consumption.* By Archibald Smith, M.D.

The various climates of Peru, as changed or modified by the measure of elevation from the sea, and other local causes, are not merely curious to the meteorologist, but more especially interesting to the physician; they are, above all, important in relation to the development in some localities, and the disappearance in others, of phthisis; this point I shall now endeavor very shortly to illustrate.

1. *What are the Localities or Climates in Peru in which Phthisis is most and least Prevalent?*—This disease is properly a product of the warm and humid valleys of the coast, such as that of the Rimac. And from Lima, where you have an extensive view up this valley, to the loftiest snow-clad peaks of the Cordillera, every gradation of climate is unfolded in the intervening distance, that one would have to pass through, in a voyage of many days, from Callao to Cape Horn. And in the inland glens beyond (as in the often-mentioned vale of Huanuco), we have noticed how the extremes of climate are brought within much nearer limits than those embraced even in this picturesque and imposing bird's eye view from Lima—especially from the bridge, looking eastward. Nor is there, in all this range of climate, a locality in which phthisis is more prevalent than in the mild and equable temperature of the capital and its immediate environs.

Piura, the most northern province of Peru, though within two or three degrees of the endless moisture and vegetation of the Equator, is yet the most hot and arid in the republic. Its maritime district is also considered the most healthy on all the coast, and remarkably free from pulmonary disease or consumption. In the pastoral sierra—viz: on the lofty slopes and colder plains of the Andes, pleurisy and pneumonia are not unfrequent; and sometimes terminate in the worst manner, by suppuration or gangrene, when left, as usually happens in remote Indian villages, without medical assistance.* Phthisis pulmonalis is, I am persuaded by a long residence in these mountain regions, little known to the native population, except as imported to the hill-land from the coast. In those warmer valleys in the centre of the Andes, where the temperature is sufficiently favorable to the growth of the banana and sugar-cane, we meet with frequent development of hepatic disease; and when the climate is particularly warm and humid, as in the province of Huamalies, on the borders of the Montaner, we even meet with severe examples of ague; and these situations are but ill calculated to restore the health of a consumptive invalid. I resided for several years in the vale of Huanuco, which—as previously mentioned—is dry, and free of malaria, at an elevation varying, as it extends along the banks of the river, from six to seven thousand feet above the level of the sea, with the themometer rarely, throughout the entire year, above 72°, or below 66° Fahr. in the shade. But this climate, though equable, did not prove favorable to the convalescence of phthisical patients brought there from other parts of the country; and I cannot say that I ever saw a case of phthisis originate in that locality. In like manner the cold—often damp and variable, and always highly rarefied—atmosphere of the mining district of Cerro Pasco, and other localities near the snow-line, is unfavorable to recovery from Phthisis. But Huarriaca, which lies in the descent from Cerro to Huanuco, is very favorable to such recovery, as I had frequent occasion to test when at the mines; whence we usually sent our patients from pulmonary affections to convalesce at this desirable place, distant eight leagues of pretty rapid descent from the silver mines of Cerro Pasco. Huarriaca is in climate very like Obrajillo, on the western slope of the

* The Indians have many native remedies for what they call *dolor de costado*, or pleurisy; but I found, in Cerro Pasco, bleeding, followed up by tartar emetic, most efficacious. In the Sierra, bleeding is better supported than on the coast, where twelve ounces of blood is a large bleeding among the white Creoles. In Lima, where the lancet cannot be used safely, tartar emetic, pushed in small doses, alone or combined with morphine, to the extent of from twelve to twenty grains, generally subdues either pleurisy or pneumonia.

Andes, and is one of those recesses in the Andine glens and defiles very productive in maize, corn or wheat, potatoes, beans, and natural pastures on the heights, as well as cultivated lucern on the straths. Such, indeed, are the marked localities, blessed with a steady temperate climate, and a dry air of about 60° Fahr. in the shade, as well as sunny cheerful sky throughout the greater part of the year. Such are the localities where phthisis proper, or tubercular disease of the lungs, is only known as an exotic!

2. *The Proportion of Deaths by Phthisis compared to other Diseases on the Coast of Peru.*—This proportion, for want of satisfactory statistical returns, can only be answered at present in respect to the capital, and even there only approximately. From data before me—let us take the mean of ten years, say from 1841 to 1850, inclusive—the average may be struck at 3,200 deaths annually, of all diseases, in Lima. Of this gross sum, the monthly hospital returns account for 1,700; while a somewhat less proportion—viz: 1,500, are indiscriminately entered in the report of the general cemetery under the title "various diseases." But from the more specific evidence as to details, furnished by the hospital reports, I will here state the average mortality for the ten years, given, in 1,700 cases yearly, as follows:

| | |
|---|---|
| From fever, | 600 |
| From dysentery and chronic diarrhœa, | 480 |
| From pleurisy and pneumonia, | 160 |
| From phthisis pulmonalis, | 320 |
| From sundry other diseases, | 140 |
| Total, | 1,700 |

Thus, next to fever and dysentery, phthisis was the most fatal disease known in Lima up to the first visitation of yellow fever in that country, from the years 1851 to 1854, as described and recorded by me in No. 203 of the "Edinburgh Medical and Surgical Journal," but with which, in our present estimate, we have nothing to do. If it can be shown in this way, that in 1,700 hospital cases of fatal termination annually, 320 of these deaths proceed from phthisis, we arrive at an average proportion for the whole mixed population of Lima admitted to hospital treatment. And further, if we put to one side the indefinite number of deaths from infantile diseases, among the 1,500 indiscriminately sent from the different parochial wards of the city, and included under one common head—viz: "various diseases;" there appears no reason why, among the remaining adult population included in the said gross parochial deaths, as distinct from the more detailed and special hospital returns, the ratio of deaths from phthisis, *as compared to other diseases*, should not be approximately the same as it is found to be in the 1,700 who died in hospital, where the proportion has been pretty reliably ascertained as above, to be about 3 in 17.

3. *In what Stage or Form of Phthisis is it found Curable by a Change from the Climate of the Coast to the Sierra?*—On the coast generally, the most usual exciting cause of pulmonary affections is observed to be some check to the perspiration (*resfrio;*) and not only pulmonary complaints, but rheumatisms, diarrhœa, and fevers acknowledge this origin. It is more particularly in spring that we see the effects of this *resfrio* in hospitals crowded with patients under the unfluence of febrile catarrh, pneumonia, pleurisy, and phthisis pulmonalis. When the frame becomes much debilitated, and especially when the patient is convalescing from some prior ailment, it is a familiar event that under these circumstances, incipient phthisis presents itself, in the form of such admonitory symptoms as growing debility, failing appetite, a slight dry cough, feverish pulse and heat, with restlessness and wakefulness by night.

In the dry and sultry summer months cases occur under a different aspect, in which, from the beginning, the gastric system is more ostensibly disordered. The tongue whitish-colored and furred; evening fever and sleepless nights; a short dry cough; depression of spirits, with a foreboding of pulmonary consumption or hæmoptysis on the part of the invalid, are so many symptoms which attend this form of attack. In all cases, whether originally of the gastric or pulmonary type, the patient or physician must not waste time in the employment of unsuccessful special remedies. And the plain reason of this practical admonition, which indeed amounts to a popular maxim in Peru, is that a change from the coast to the mountain climates, graduated as the case may require, will do more to restore health than all the drugs within their ken; and that, if this easy migration be too long deferred, confirmed as well as hopeless phthisis will be the end of disorders so initiated on the coast.

But though it be here necessary to characterize such examples as the above, in pointing out the introductory forms which phthisis assumes in Peru; yet it is important to bear distinctly in mind, that the most common prelude, as well as attendant, of the Lima phthisis pulmonalis, undoubtedly is hæmoptysis; to which there appears to be a remarkable predisposition among all the mixed classes and races of the population, particularly in the white, Creole, and brown females of preponderating Indian caste. The healthy, full-chested, mountain Indian mother, if engaged in the maternal duty of suckling her young on the coast, often acquires a predisposition to hæmoptysis, to which she has shown no tendency whatever so long as she lived and nursed on the mountains. On the hill-land the ordinary functions of the digestive organs are vigorously exercised; while on the coast, the long-continued influence of a warm and humid atmosphere not only keeps up a relaxation of the skin, but induces a more languid appetite, and a less perfect and healthy action of the stomach and bowels, &c., which soon tells on the whole system. Europeans soon become lazy, and unwilling to take exercise on foot, in the Lima climate, and suffer a great, though gradual, loss of nervous and muscular power. The offspring, especially the male offspring, of the athletic Spaniard, grows up a comparatively delicate man; but the negro race thrive well on the coast, and retain the muscular power of their progenitors. The white family always suffer more or less from a protracted residence in Lima, where congestive diseases are sure to arise in this race; and more particularly the prevailing disorders, hæmorrhoids, blennorrhœa, dysentery, &c.

Whenever hæmoptysis shows itself in Lima—which it often does in the Creole ladies after an evening party (*tertulla*), without any previously perceived sign or suspicion of so great a misfortune—the circumstance is always one of alarm.

The spitting of blood may be very slight at first, and attended with a slight cough; and from so apparently simple a beginning, experience and common observation lead the patient, friends, and physicians together to fear the approach of phthisis, unless the hæmoptysis and cough can be speedily subdued. As a general rule, in such cases, phthisis is always suspected to lurk in the background, unless its incubation be promptly checked by a change of climate. The ordinary result is, that those so circumstanced, especially when of the delicately-organized, fair, Creole race, very rarely trust to medicine or to the assistance of the physician, but at once order the mules and other necessary arrangements for a journey to the interior. It is only by this decided conduct that they hope permanently to guard against a future and more formidable return of hæmoptysis, with its phthisical consequences; and they seek at first notice of the disease to insure a full reparation of the injured respiratory organs, by an adequate continuance in the well-known and appropriated regions of convalescence.

When cases thus inaugurated—which are far too frequent in Lima and other parts of the coast of Peru—go on for a few weeks, not to say months, without decided amendment under medical treatment, we may expect to find on examination positive signs of pulmonary consumption. Now, then, besides occasional returns of hæmoptysis more or less developed, varying from colored sanguineous sputa, to mouthfuls or even cupfuls of blood at a time, there is also more or less cough, soon attended by some degree of pain in the chest; depression of spirits, failure of appetite, with loss of flesh, and lassitude; some notable change in the respiratory sound, or perceptible deviation from the normal murmur, with almost always obscurity of sound on percussion under either the right or the left clavicles. No Lima junta of experienced native or well-acclimated European physicians, would for a moment hesitate to order to the sierra a patient in the condition I have just described. They would deem this transfer of climate as the only security for the patient.

Under such conditions I have witnessed the application of all approved European remedies of every school fully tried, where the phthisical patient was, for one reason or another, destined to run his course on the coast and in the capital, under the eye of able assistants, but always with the same fatal termination.

Cod-liver oil, extensively used of late years, has appeared to alleviate the pulmonary symptoms, by improving the habitual state of the digestive organs, but, as far as I know, it did no more in that country, whatever may have been its success in Europe.

I have sometimes seen cases of pneumonia, imperfectly cured, terminate in chronic phthisis, on the coast of Peru. I have also met with cases of passive and chronic hæmoptysis sustained by pulmonary congestion, or consequent upon heart disease, which never passed into phthisis. But such cases are easily distinguished for the most part, and I may just say in passing, that small doses of spirits of turpentine—say twenty drops thrice a day—have been useful in stopping these passive forms of pulmonary hæmorrhage.

In advanced stages of phthisis, attended with opaque and purulent sputa, colliquative sweats, bronchial and cavernous respiration, with all the aggravated symptoms of hectic fever—even in such a plight, the change from the climate of Lima or the coast to that of the Andine slopes (at modern elevations relatively to the snow line) has been known to prolong life for years, and allow the patient renewed strength to return from time to time to the coast, with marked improvement in general health, as well as in the condition of the lungs, and quite free from fever. But after a few years, such partial convalescents have succumbed to a fresh accession from cold or other exciting cause. But while I state these facts, and could cite individual instances in point, it should never be forgotten that the timeous removal to the sierra is intended to prevent the advancement of phthisis beyond its first initiatory stage in the hæmoptoic form of invasion so prevalent in Peru, or to cause it to retrograde altogether, even from this primary condition. It must be clearly understood, therefore, that I claim the curative effects of the Andine climates, on the broadest grounds of facts and experience, in favor of the early stage only, and not the more advanced periods of pulmonary consumption, when there is, correctly speaking, no sound lung to rescue.

4. *What are the Inland Localities in Peru approved as the Best for Convalescence from Phthisis ?*—I shall speak of the localities best known in, and most convenient to, the capital; other inland positions of corresponding temperature will naturally be resorted to from other points of the coast, according to their contiguity. On the Pacific coast of the Corderilla, and by the Pasco road from Lima, Haraway (usually pronounced Yaraway) and Canta are considered the best localities; and Huamantanga is also considered favorable; but Canta, above all, on this route, is allowed to be most desirable, being about twenty-five leagues from Lima, and at an elevation of 10,000 feet, on a height overlooking Obrajillo, which latter is in a hollow locked in by hills, and about 1,000 feet lower than Canta. Again, by the Zarma road from Lima, Matucana and San Mateo are favorable climates; the former, according to McLean, is 8,026, and the latter 10,984 feet high; and of the two, Matucana is considered the best. But Canta is found preferable to either as a place of permanent convalescence. Cullua, enclosed in a basin-shaped hollow a few leagues above Obrajillo, on the Pasco road, is 12,000 feet above the sea, and corresponds in climate with Chicla, a few leagues above San Mateo on the Zarma road, and at an elevation of above 12,000 feet, according to McLean and Herndon's reckoning. Both these localities are hostile to the phthisical patient.

When it is determined to pass the Cordillera for convalescence this is usually done by the pass of Yauli or by Tucto, to the temperate valleys of Zarma, Jauja, and Huancayo. The elevation at the pass of the Viuda mountain above Culluay on the one hand, and of that of Antarangra (also called Antocona) above Chicla on the other, is nearly equal, as far as can be determined by the measurements of different observers. McLean gievs the one at 15,543, and Rivero the other—viz., that of the Viuda, at 15,500; the Viuda being 15,868 feet—just 1,000 feet above the line of glaciers or permanent snow. Across the Cordillera gates or passes (Portachuelas), the patient, if very weak, is conveyed in a litter, and if his direction be Pasco, he cannot remain there, but must at once pass through to Huarriaca, a climate quite analogous in temperature to that of Obrajillo, only with better ventilation. But physicians from Lima always send their phthisical patients (when ordered across the Cordillera) to Zarma and Jauja as the great sites of convalescence; and on the way to these celebrated localities, Matucana is the favorite resting place of phthisical and hæmoptoic patients. It is at this point, in the headland of the valley of the Rimac, enjoying a mild atmosphere on the confines of the air of the coast and the sierra, and just within the rain line, without being yet too wet or cold, that the invalids alluded to receive the first kindly impressions of improving health, and after a longer or shorter stay here, proceeded to those more favorable climates, in higher elevations, beyond the first Cordillera.

I should state expressly, that the extensive valley of Jauja, rather cooler in temperature, and also of a few hundred feet more elevation above the sea than Zarma (which Herndon gives at 9,738), is allowed to have a decided superiority for the recovery of the hæmoptoic and phthisical ivalid. The climate of this locality is temperate, and productive of a great variety of grain and green crops. But for the cure of phthisis, the Montana climate, for at least eight months in the year, is too damp, and if the patient be not careful in ordinary ablution—which natives prefer doing when the sun shines—the body is apt to be chilled. Lieutenant Herndon experienced this effect after bathing, and cautions his readers on the subject.

I shall conclude these cbservations by endeavoring to solve an important problem bearing intimate reference to our present inquiry, and which I find suggested in Dr. James Copland's very elaborate and instructive article on Tubercular Phthisis, recently published in Part 17 of his valuable 'Dictionary of Practical Medicine.'

The problem I allude to is contained in the following extract:—

"Having ascertained the frequency of the disease in the aborigines of a country or climate, it is next of importance to know how far that frequency may be modified, diminished, or increased by change to other countries, either colder or warmer, or of higher or lower elevation, etc., and by the adoption of different food and other habits.'" [p. 1130, sect. 205.]

I beg the reader's attention to this quotation. I hope I may, without any undue pretension, be allowed to say that I feel not only authorized, but called upon as a matter of duty to record on this head the result of my long experience in different climates of Peru. I shall therefore remark that, as regards the native white Creole and the brown races of mixed blood, this problem may be considered as solved in cases of incipient phthisis pulmonalis attended with more or less hæmoptysis. By change to other countries—for example, to Chile, which is colder, or to Guaquil, which is on the *Equator*, and consequently warmer—the effect on the patient from Lima has been so often tried and found injurious, that this is a change of climate which no experienced resident physician would venture to recommend. But by proceeding inland to the valley of Jauja, at the elevation of ten thousand feet above the sea, such incipient phthisical cases—especially of the hæmoptoic type, as I have defined—are always relieved, and almost always cured, provided the patient remain long enough in the uplands to insure this result.

Time is required to bring about a radical organic change, for when individuals apparently quite recovered in Jauja descend to the coast, and particularly to the capital, within a few months the hæmoptic and other phthisical symptoms have been observed to return, rendering a longer residence in the sierra necessary to insure a permanent cure. A year's sojourn in the sanitary climate of the hill-land is usually considered indispensable in serious cases, which have demanded a transandine climate.

Milder cases and slighter indications of pulmonary disease, with tubercular development, often yield to a few months' residence at Matucana, Haraway, or Canta, on the western slope of the Andes, and not far from the resources of the capital.

The unvarying experience of centuries, perfectly relied upon by the natives, proclaims this change from the coast to the sierra climates, to afford undoubted beneficial results to the native white, as well as the diverse shades of brown and olive races of the coast, when laboring under hæmoptysis or pulmonary consumption. The negro is less subject to phthisis, and also reluctant to encounter the bracing air of the Cordillera; his favorite element is the warm and humid air of the coast. The influence of race in the cure of disease is wisely considered by Dr. Copland as of greater importance than has yet been bestowed upon it. In Peru, I found this truth constantly illustrated in practice. For instance, in dysentery, calomel and opium properly and timeously administered, are almost infallible in the Indian race, in the white far less certain, and in the negro cannot be depended upon at all. In yellow fever, turpentine cured as many as fifty per cent. of Indians, apparently in a hopeless condition, being sent to the Lazaretto, as it was believed, in an incurable state; but in the whites, turpentine, as administered by us in Peru in the year 1854 was of comparatively little power; and as for the negroes, we had no opportunity of ascertaining its effects, since in them this malady, so fatal to the whites, was scarcely experienced, except as a slight fever with headache, which by the aid of common enemata passed off in a few days, leaving no bad symptom or dregs of disease behind it.

But as regards phthisis, which we have been considering above, I have always seen cases of the character described by me turn out well through migration to the sierra. And I may truly say, that from my own long experience in Peru, and knowlege of these cases, I could easily recount a multitude of permanent cures, also familiar to many native physicians.

This result, as far as the natives are concerned—a goodly mixture and variety of races, we must admit—is simply conclusive matter-of-fact; and now that the communication by Panama is so easy, it may be worth while to test the effects of the Andine climate of Peru on the European phthisical invalid. I had little opportunity to do so with the English under my charge in that country, but as far as my experience went, it was as favorable to the European as to the native Creole. But allowances, no doubt, must be made for different habits of life and other causes. The benefit received by Peruvians in the instances in question are too evident to admit of cavil, nor can the good effects be explained on the score of mere change of scenery and the pleasures of traveling All coast-born Peruvians leave the neighborhood of the sea and their native towns—above all, Lima—with extreme reluctance, and look upon sierra as a kind of Siberia—a place of privation and exile. But in spite of all these prejudices and dislikes, when they realize the change of the sierra, they are constantly seen to recover there, under conditions of pulmonary tubercular disease which would undoubtedly terminate fatally, and that very soon, on the coast.

On the mountains, the Limenian habits of diet are necessarily somewhat changed, and the invalids are naturally led to more exercise in the open air; but yet their in-door habits, with their gambling propensities, will ever predominate, whether on the hills or coast. Cards and dice, indeed, are esteemed not merely an amusement, but an indispensable part of a genteel education.

The air of the mountains—in those elevated localities pointed to as suitable to the recovery of the phthisical invalid—is free from the malaria of the coast, and (as we have already learnt) clear, light, cool, and invigorating—alike removed from the extremes of cold or heat, and, upon the whole, remarkably equable.

On the coast, they continually drink in abundance cooling acidulated beverages, as lemonades, pineades, &c., and the classes in better circumstances (under the idea that a weakening climate needs strengthening food) use much more animal food than a climate so mild, with an indolent life, would seem to require. Indeed, all grades of the population consume great quantities of lard and pork, and also of fish fried in pans of boiling lard. This kind of cooking goes on in the open squares, corners of streets, and market-places, every evening and morning, for the convenience of the populace or lower classes, who thus feed in the open air at small cost of money and free from domestic trouble. Sweets, pastry, and fruits they eat at all hours, irrespective of their regular meals. On the removal of invalids from such a population to the sierra, the same facilities do not offer. The mountain diet is necessarily more simple, and the habits of life there assumed for the time, are more in unison with those of the rural population of the district.

